# Decision Analysis of Manufacturer-Led Closed-Loop Supply Chain Considering Corporate Social Responsibility

**DOI:** 10.3390/ijerph192215189

**Published:** 2022-11-17

**Authors:** Qi Zhang, Yong Liu, Zhiyang Liu

**Affiliations:** School of Business, Jiangnan University, Wuxi 214122, China

**Keywords:** corporate social responsibility (CSR), decision analysis, coordination mechanism, two-part tariff

## Abstract

With the rapid development of the economy, a growing number of consumers and enterprises are paying attention to corporate social responsibility (CSR). Meanwhile, there exist a variety of conflicts in closed-loop supply chain management. To analyse and deal with the decision problems of the manufacturer-led closed-loop supply chain with CSR, by using the manufacturer Stackelberg game, we construct some basic models considering CSR, and exploit them to analyse the optimal decisions of supply chains with and without CSR under centralized and decentralized decision making and explore the influence of CSR on supply chain, and then we establish a coordination mechanism through two-part tariff.

## 1. Introduction

The rapid development of the economy has not only enhanced the strength of enterprises, but also improved people’s living standards. However, the phenomenon of unbalanced economic development and social development follows on from this as a consequence. Problems such as large emissions of greenhouse gases, frequent smog, waste of resources and inadequate protection of workers’ rights and profits are becoming increasingly serious. The closed-loop supply chain (CLSC) was put forward in 2003, referring to the complete supply chain cycle of an enterprise from purchase to final sale, including the reverse logistics of product recovery and life cycle support. The closed-loop supply chain development mode can promote the cost optimization of enterprises, while also following the green development and circular development. At the same time, corporate social responsibility (CSR) has gradually entered the public eye. Good CSR performance brings better economic benefits and reputation. In real life, more and more enterprises are attaching importance to corporate social responsibility, and actively participate in the construction of CSR. As a well-known manufacturer, Hong Qi has set up the Poverty Alleviation Dream Fund and built five Red Flag Dream Wisdom Schools. At the same time, Hong Qi adopts the most stringent environmental protection material selection standards, strictly selects the instrument panel, door guard panel and other component materials, and carries out production and processing in accordance with the national environmental protection standards. Hong Qi effectively recycles materials before and after manufacturing, and also picks up old cars and reuses them. Most of Hong Qi’s models have more than 10 environmental protection patents, with the new Hong Qi H5 vehicle bringing the number of environmental protection patents up to 23. It actively fulfils its corporate social responsibility to enhance its reputation while promoting economic and social development. From the perspective of the whole system, it is of great practical significance to clearly classify the relationship between CSR undertakings and enterprise decisions and the profits of the closed-loop supply chain. With the input of CSR, enterprise costs will inevitably increase. Whether to fulfil corporate social responsibility and how to fulfil it to maximize the benefits of the supply chain system have become urgent problems for enterprise managers to solve. In a manufacturer-led closed-loop supply chain, due to information asymmetry and uncertain demand, upstream and downstream enterprises make decentralized decisions for their own profits, resulting in the loss of the overall profits of the supply chain, so it is greatly important to design a coordination mechanism to alleviate the conflict. In view of this, we discuss the optimal decisions of the supply chain and explore the influence of CSR on the supply chain, and establish a two-part tariff coordination mechanism. In addition, different from other existing literature, this paper considers the cost and profit brought by CSR activities in the objective function, and quantifies the actual factors into the coordination of CSR in the closed-loop supply chain. This paper encourages all enterprises to actively undertake social responsibility and embed it in the optimization of supply chain cooperation, which has strong management implications for enterprises.

The remainder of the paper is organized as follows. Section 2 reviews the related literature. In Section 3, we discuss and present the optimal decisions of the closed-loop supply chain with and without CSR under decentralized and centralized making, and give a coordination mechanism. A numerical experiment is performed in Section 4. Section 5 describes the management implications. Finally, in Section 6, we draw key conclusions and point out some defects.

## 2. Related Work

The development of economic globalization has not only greatly enriched materials, but also brought problems such as resource waste and environmental pollution. In terms of enterprises, the closed-loop supply chain includes green development, environmental protection and sustainable development, which can be collectively referred to as corporate social responsibility. Focusing on the research target of this paper, we will discuss the existing aspects of corporate social responsibility, the closed-loop supply chain, the combination of the two, and their application in the manufacturer-led closed-loop supply chain.

With the growing development of the industrial supply chain, global governments are also paying more attention to all kinds of environmental issues, and the concept of environmental protection has been introduced into the supply chain. First, the role of government in green supply chain management has become particularly important. By proposing a green supply chain model with a duopoly structure, Barman analysed how government subsidies for green products and tax policies for non-green products affect the profitability of supply chain members [1]. Barman further researched optimal pricing strategies with or without government subsidy, aiming to maximize the overall profit of the supply chain [2]. On the other hand, for enterprises, environmental protection is embodied through the concept of corporate social responsibility, so corporate social responsibility has attracted a great deal of attention from the public and scholars. Some scholars have explored how to carry out the operation coordination [3], allocation [4], investment and relevant decisions [5] of corporate social responsibility, and summarized its impact on industry [6,7,8]. Andrews, Davis and Blomstrom, and Huse proposed a framework for defining and regulating corporate social responsibility [9,10,11]. Goering analysed the simple linear demand bilateral monopoly and proposed a marketing chain coordination model of corporate social responsibility [12]. Hosseini et al. found that wholesale price contracts based on compensation could motivate retailers to increase their CSR input [13]. Lin et al. built a manufacturer-led green supply chain model to discuss the impact of demand disturbance and retailers’ CSR behaviour on green supply chain decisions, and studied the coordination of the green supply chain [14].

The closed-loop supply chain model is widely favoured by enterprises, and it has aroused a large wave of research on its coordination mechanisms. Fleischmann designed a logistics network for a supply chain from the reverse perspective, creating the earliest definition of the closed-loop supply chain [15]. In fact, the emergence of the closed-loop supply chain is closely related to the development of the economy and society. In terms of product pricing, Ma et al. studied the impact of consumer subsidies on product pricing strategies in a dual-channel closed-loop supply chain [16]. When discussing the competition between OEM and IO in product sales and recycling, Bulmus et al. found that the acquisition price of OEM only depended on its own cost structure, not on the acquisition price of IO [17]. In terms of information symmetry and asymmetry, after Pasternack put forward the supply chain contract theory in the early stage, the research on supply chain contracts in academia and industry developed rapidly [18]. Zhang et al. studied the design of a retailer’s optimal two-part tariff contract in a closed-loop supply chain in which the manufacturer carried out recycling and re-manufacturing, and where the recovery cost information was asymmetric, and discussed the nature of the contract and the impact of the recovery cost information asymmetry on the entire closed-loop supply chain [19]. In terms of the uncertainty of demand, Li et al. constructed the total cost model of pricing before re-manufacturing under the assumption of random fuzzy demand for the uncertainty of the re-manufacturing process [20]. Liao et al. constructed a re-manufacturing closed-loop supply chain game model with centralized and decentralized decision making guaranteed by retailers by using the alternative two-product newspaper boy model (RS-CLSC, Closed-Loop Supply Chain of Servicing) [21]. In addition, the problem of product recycling and re-manufacturing between the upstream and downstream of the supply chain has also aroused discussion among scholars. Takamichi et al. studied the dynamics of first-order auto-regressive demand and reverse recovery process of the closed-loop supply chain [22]. In addition, Barman et al. presented a production inventory system with a manufacturer–retailer supply chain, and analysed the shortcomings of the two-tier supply chain model [23].

While studying the theories related to corporate social responsibility and supply chain, scholars have gradually linked corporate social responsibility with decisions related to product design and pricing in the closed-loop supply chain. Hsueh, Modak, and Ni et al. studied the interaction between CSR and daily operation of enterprises, and proposed the positive role of CSR in supply chain enterprises [24,25,26]. Panda started from practical problems and explored effective solutions to channel conflicts and supply chain profit distribution by introducing corporate social responsibility [27,28]. Hsueh proposed that new profit-sharing contracts can improve the performance of the corporate social responsibility supply chain [29]. Modak et al. studied the three-stage supply chain composed of the manufacturer, multiple distributors and multiple retailers and only the manufacturer undertakes CSR, and proposed a new profit-sharing contract to coordinate the three-stage supply chain. They also studied a two-stage supply chain consisting of the manufacturer and two competitive retailers and only the manufacturer assumes CSR, and proved that the two-part tariff contract can effectively reduce and even solve supply chain conflicts [30,31]. Wu et al. introduced two kinds of flexible quantity discount contract and wholesale price contract to deal with the problem of the manufacturer’s profits reduction caused by the lack of social responsibility efforts of suppliers, and proved that these two kinds of contracts can significantly improve the performance of supply chain [32]. Giovanni established a recycling place for used batteries to make consumers feel satisfied with participating in environmental protection in the process of recycling, and in this kind of CSR behaviour, a joint maximization incentive mechanism was designed to encourage manufacturers to increase green investment, so as to improve the benefits of the closed-loop supply chain [33].

Corporate social responsibility not only includes social welfare and labour rights, but also closely affects the daily production and operation of enterprises. In the manufacturer-led supply chain research, Hosseini-Motlagh et al. studied the closed-loop supply chain formed by two competing manufacturers and one retailer, and proposed a wholesale pricing mechanism based on compensation to improve recycling efficiency and overall operation performance [13]. Wu et al. built a two-stage closed-loop supply chain model of environmental protection, and found that the recycling situation between manufacturers and retailers was mainly determined by the transfer price, and it was unfavourable for the third party to dominate recycling [34]. Some scholars like to add realistic factors into the consideration of CSR. For example, Modak et al. took donations into consideration and developed the best closed solution for three decentralized and centralized channel structures [35]. Wang et al. considered the role of government subsidies in the closed-loop supply chain and built a functional model to understand the influence of government subsidies, corporate social responsibility coefficient and equity issues on decisions [36].

In light of the above discussion and analysis, our research differs from the existing literature in three main aspects: (1) The existing literature mainly analyses the impact of social responsibility in the performance of the closed-loop supply chain, but does not encourage all enterprises to actively undertake social responsibility and embed it into the optimization of supply chain cooperative relationship. In addition, costs and profits brought by CSR activities are rarely considered in the objective function, and few studies have quantified practical factors into the coordination of CSR in the closed-loop supply chain. (2) Most previous studies focus on the mutual game of nodal enterprises such as retailers, manufacturers, or suppliers with respect to their own profits, without considering the impact of supply chain members’ behaviours. (3) The current research on closed-loop supply chain coordination considering CSR is based on an idealized state, and realistic factors such as demand uncertainty and information asymmetry are not fully considered. To deal with the shortcomings of the existing literature, by taking CSR into consideration in the manufacturer-led closed-loop supply chain, we establish some basic models considering CSR, and discuss the optimal decisions of the supply chain, and analyse the influence of social responsibility level on the decision of supply chain members and the relationship between demand and cost information and decision variables, and then we propose a two-part tariff contract mechanism.

## 3. Decision Analysis and Coordination of Supply Chain with CSR

At present, corporate social responsibility is constantly attracting the attention of enterprises, especially manufacturers. For example, in 2020, when COVID-19 broke out, Mengniu, the State Grid, the Yufa Group, and other companies actively responded to the call of party committees and governments at all levels, donating money to support the front line, which not only provided strong support for the victory in epidemic prevention and control, but also resulted in them obtaining a much better reputation and achieving sustainable development. However, the input of CSR can cause conflicts between manufacturers and retailers in the closed-loop supply chain. As a result, based on a closed-loop supply chain with manufacturers as channel leaders and product recyclers, and retailers as the market end, we introduce corporate social responsibility, starting from centralized decisions and decentralized decision making, and explore the relationship between the closed-loop supply chain and corporate social responsibility by constructing mathematical functions. Then, we solve the optimal decisions of supply chain members and propose the corresponding coordination mechanism.

### 3.1. Some Basic Models

Before building some basic models, we make the following assumptions, and the description of the parameters is shown in Table 1.

**Hypothesis 1.**  *Suppose there exists a closed-loop supply chain consisting of a dominant manufacturer and a retailer that are risk neutral. In addition, scrap product re-manufacturing is carried out, and then these re-manufactured products are sold to retailers, and then they are resold by retailers to consumers. Let* w,p *stand for the wholesale price of the manufacturer and the retail price of the product of the retailer, respectively.*

**Hypothesis 2.**  *Assume that the market demand is positive. According to the relevant literature of Hsueh* [24] *and Modak* [25] *et al., let* D *express the market demand function, which satisfies* D=a−bp. *Among this,* a *is the market size,* b *is the price sensitivity coefficient. At the same time, let* $C_{L},\xi ,\beta$ *represent recovery scale parameter, CSR cost coefficient and the sensitivity coefficient, respectively, and* a>0, b>0, $C_{L}>0$, ξ>0, β>0*. Additionally,* a *is much bigger than the other parameters, and* $C_{L}>\frac{\Delta^{2}b}{2},b>\beta$.

**Hypothesis 3.**  *In the reverse recycling chain, the manufacturer recycles the waste products by relevant means, which is similar to Savaskan’s recycling and re-manufacturing of waste products led by manufacturers* [37]*. Let* $c_{m},c_{r},\tau ,\Delta ,C(\tau ),C_{L}$ *express the unit cost of the new product, unit cost of the re-manufactured product, recovery rate of waste products, average production cost of manufacturers, recovery cost and scale parameter, respectively. In addition, they satisfy* 0≤τ≤1*,* $\Delta =c_{m}-c_{r}$*,* $\tilde{c}=c_{m}-\Delta \tau$, $C(\tau )=\frac{1}{2}C_{L}\tau^{2}$*, and* $C_{L}>\frac{\Delta b\xi (a+b\Delta -bc_{m})}{2(b\xi -\beta^{2})}$.

**Hypothesis 4.**  *Corporate social responsibility involves environmental protection, labour rights, resource conservation, etc. To explore and consider the impact of corporate social responsibility on the decisions of members in the closed-loop supply chain, for the purpose of facilitating calculation and comparison, we abstract corporate social responsibility expressed as*$e_{m}$*, and then the corresponding CSR input cost is*$C(e_{m})=\frac{1}{2}\xi e_{m}{}^{2}$*. In addition, considering the input of CSR, the original demand function will be written as*$D=a-bp+\beta e_{m}$.

**Hypothesis 5.**  *Suppose that there is only an operation of a single cycle, that is, both the reverse and forward processes are completed within one cycle. Therefore, let* $\pi_{m},\pi_{r},\pi$ *represent the profits of the manufacturer, retailer, and supply chain, respectively. According to the above hypothesis and analysis, the expressions can be written as follows:*

(1) $$\pi_{m}=(w-\tilde{c})D-C(e_{m})-C(\tau )=(w-c_{m}+\Delta \tau )(a-bp+\beta e_{m})-\frac{1}{2}\xi e_{m}{}^{2}-\frac{1}{2}C_{L}\tau^{2}$$

(2) $$\pi_{r}=(p-w)D-C(e_{m})=(p-w)(a-bp+\beta e_{m})$$

(3) $$\pi =[a-bp+\beta e_{m}](p-c_{m}+\Delta \tau )-\frac{1}{2}\xi e_{m}{}^{2}-\frac{1}{2}C_{L}\tau^{2}$$

### 3.2. Decision Analysis of Manufacturer-Led Closed-Loop Supply Chain without CSR

In this section, we mainly discuss the decisions of the supply chain without CSR (namely, $e_{m}=0$) under decentralized and centralized decision making, and the profit expressions of the manufacturer, retailer and supply chain, respectively, can be written as follows:

(4) $$\pi_{{}_{r}}^{N}=(p-w)(a-bp)$$

(5) $$\pi_{{}_{m}}^{N}=(w-c_{m}+\Delta \tau )(a-bp)-\frac{1}{2}C_{L}\tau^{2}$$

(6) $$\pi^{N}=(p-c_{m}+\Delta \tau )(a-bp)-\frac{1}{2}C_{L}\tau^{2}$$

In the supply chain without CSR, firstly, based on the price decision of the retailer, the dominant manufacturer sets the wholesale price and the recovery rate according to its own profits, then the retailer makes a decision and sets the retail price based on the price of the manufacturer and its own profits, so that p,w,τ can be determined. They follow the manufacturer Stackelberg game.

#### 3.2.1. Optimal Decisions without CSR under Decentralized Decision Making

Under decentralized decision making, both the retailer and the manufacturer make relevant decisions to maximize their own profits. Here, some theorems and conclusions are given.

**Theorem 1.**  *In the supply chain without CSR, let* $w_{N}^{d*},p_{N}^{d*},\tau_{N}^{d*}$ *represent the optimal wholesale price, the optimal retail channel price, and the optimal manufacturer recovery rate under decentralized decision making, respectively. If there exist unique values to maximize the supply chain members, the following will hold true:*

(7) $$w_{{}^{N}}^{d*}=\frac{\Delta^{2}ab-2bC_{L}c_{m}-2aC_{L}}{b(\Delta^{2}b-4C_{L})}$$

(8) $$p_{N}^{{}^{d*}}=\frac{\Delta^{2}ab-bC_{L}c_{m}-3aC_{L}}{b(\Delta^{2}b-4C_{L})}$$

(9) $$\tau_{N}^{{}^{d*}}=-\frac{\Delta (a-bc_{m})}{\Delta^{2}b-4C_{L}}$$

The proof is shown in Appendix A. 

Let $D_{N}^{d^{*}}$ express the optimal demand; by substituting the above results $p^{d*}$ into $D_{N}^{}$, we can obtain the optimal quantity $D_{{}_{N}}^{d*}=-\frac{C_{L}(a-bc_{m})}{\Delta^{2}b-4C_{L}}$. Assume $\pi_{m_{}}^{Nd*},\pi_{r_{}}^{Nd*},\pi_{N}^{d*}$ as the profits of the manufacturer, the retailer, and the whole supply chain after making the optimal decision under decentralized decision making. By putting the above optimal values into Equations (4)–(6), respectively, we can obtain their maximization profits as follows:

(10) $$\left\{\begin{array}{l} \pi_{m}^{Nd*}=-\frac{1}{2}\frac{C_{L}{\left(a-bc_{m}\right)}^{2}}{b(\Delta^{2}b-4C_{L})}\\ \pi_{r}^{Nd*}=\frac{C_{L}{}^{2}{\left(a-bc_{m}\right)}^{2}}{b{\left(\Delta^{2}b-4C_{L}\right)}^{2}}\\ \pi_{N}^{d*}=-\frac{1}{2}\frac{C_{L}{\left(a-bc_{m}\right)}^{2}(\Delta^{2}b-6C_{L})}{b{\left(\Delta^{2}b-4C_{L}\right)}^{2}} \end{array}\right.$$

By calculation, we obtain $\pi_{m}^{Nd*}>\pi_{r_{}}^{Nd*}$. This shows that in the closed-loop supply chain, when the manufacturer first sets the corresponding price based on its own considerations, the retailer must consider its own and market factors. If the adjustment space is small, the retailer will gain less profit than the manufacturer.

#### 3.2.2. Optimal Decisions without CSR under Centralized Decision Making

Under centralized decision making, the supply chain system will pursue the maximization of the profits of the whole supply chain. According to Equation (6) and on the basis of the facts and the principle of derivation, we can calculate $p_{N}^{c*}$ and $\tau_{N}^{c*}$ under centralized decision making.

**Theorem 2.**  *In the supply chain without CSR, let* $p_{N}^{c*},\tau_{N}^{c*}$ *stand for the optimal price and optimal recovery of the supply chain, respectively, under centralized decision making. If there exists a unique value for maximizing the supply chain, the following will hold true:*

(11) $$p_{N}^{c*}=\frac{\Delta^{2}ab-bC_{L}c_{m}-aC_{L}}{b(\Delta^{2}b-2C_{L})}$$

(12) $$\tau_{N}^{c^{*}}=-\frac{\Delta (a-bc_{m})}{\Delta^{2}b-2C_{L}}$$

The proof is shown in Appendix A.

According to the theorem, let $D_{N}^{c*}$ stand for the optimal demand; by putting the above two optimal solutions into the demand function, we can determine its value:$D_{N}^{c*}=-\frac{C_{L}(a-bc_{m})}{\Delta^{2}b-2C_{L}}$. Let $\pi_{N}^{c*}$ be the optimal profits of the whole supply chain under centralized decision making; by substituting Equations (11) and (12) into Equation (6), we can obtain the optimal profits:

(13) $$\pi_{N}^{c*}=-\frac{1}{2}\frac{(b^{2}c_{m}{}^{2}-2abc_{m}+a^{2})C_{L}}{b(\Delta^{2}b-2C_{L})}$$

### 3.3. Decision Analysis of Manufacturer-Led Closed-Loop Supply Chain Decision Model with CSR

In this section, corporate social responsibility in the closed-loop supply chain will be considered for comparison. As assumed and analysed above, the profit function of the manufacturer and the retailer are as follows:

(14) $$\pi_{m}=(w-\tilde{c})D-C(e_{m})-C(\tau )=(w-c_{m}+\Delta \tau )(a-bp+\beta e_{m})-\frac{1}{2}\xi e_{m}{}^{2}-\frac{1}{2}C_{L}\tau^{2}$$

(15) $$\pi_{r}=(p-w)D-C(e_{r})=(p-w)(a-bp+\beta e_{m})$$

#### 3.3.1. Optimal Decisions with CSR under Decentralized Decision Making

In the supply chain with CSR, the dominant manufacturer and retailer follow the Stackelberg game. Let $\pi_{{}_{m}}^{d},\pi_{{}_{r}}^{d}$ stand for the profits of the manufacturer and retailer, respectively. Then, we can obtain a decentralized decision making model as follows:

(16) $$\pi_{{}_{m}}^{d}=(w-\tilde{c})D-C(e_{m})-C(\tau )=(w-c_{m}+\Delta \tau )(a-bp+\beta e_{m})-\frac{1}{2}\xi e_{m}{}^{2}-\frac{1}{2}C_{L}\tau^{2}$$

(17) $$\pi_{{}_{r}}^{d}=(p-w)D-C(e_{r})=(p-w)(a-bp+\beta e_{m})$$

**Theorem 3.**  *In the supply chain with CSR, let* $p^{d}{}^{*},\tau^{d*},w^{d*},e_{m}{{}^{d}}^{*}$ *express the optimal retail price, recovery rate, wholesale price, and optimal CSR level, respectively, under decentralized decision making. If there exists a unique value to maximize the profits of the supply chain members, the following will hold true:*

(18) $$p^{d}{}^{*}=\frac{[(-bc_{m}-3a)\xi +\beta^{2}c_{m}]C_{L}+\Delta^{2}ab\xi }{(\beta^{2}-4b\xi )C_{L}+\Delta^{2}b^{2}\xi }$$

(19) $$\tau^{d*}=-\frac{\Delta b\xi (a-bc_{m})}{\Delta^{2}b^{2}\xi -4b\xi C_{L}+\beta^{2}C_{L}}$$

(20) $$w^{d*}=\frac{(-2bc_{m}\xi -2a\xi +\beta^{2}c_{m})C_{L}+\Delta^{2}ba\xi }{(-4b\xi C_{L}+\beta^{2}C_{L})+\Delta^{2}b^{2}\xi }$$

(21) $$e_{m}{{}^{d}}^{*}=-\frac{\beta C_{L}(a-bc_{m})}{(\beta^{2}-4b\xi )C_{L}+\Delta^{2}b^{2}\xi }$$

The proof is shown in Appendix A.

According to Theorem 3, in the supply chain with CSR, by putting the above optimal solutions into the demand function under decentralized decision making, we can determine the optimal demand as follows:

(22) $$D^{d^{*}}=-\frac{b\xi C_{L}(a-bc_{m})]}{\Delta^{2}b^{2}\xi -4b\xi C_{L}+\beta^{2}C_{L}}$$

Let $\pi_{m_{}}^{d*},\pi_{r_{}}^{d*},\pi_{}^{d*}$ express the profits of the manufacturer, the retailer, and the whole supply chain, respectively, after making the best choice under decentralized decision making. Then, we can obtain the final solutions as follows:

(23) $$\pi_{r}{}^{d*}=\frac{b\xi^{2}C_{L}{}^{2}{\left(a-bc_{m}\right)}^{2}}{{\left(\Delta^{2}b^{2}\xi -4b\xi C_{L}+\beta^{2}C_{L}\right)}^{2}}$$

(24) $$\pi_{m}{}^{d*}=-\frac{{\left(a-bc_{m}\right)}^{2}\xi C_{L}}{2\Delta^{2}b^{2}\xi +2\beta^{2}C_{L}-8b\xi C_{L}}$$

(25) $$\pi_{}{}^{d*}=-\frac{1}{2}\frac{{\left(a-bc_{m}\right)}^{2}\xi C_{L}(\Delta^{2}b^{2}\xi +\beta^{2}C_{L}-6b\xi C_{L})}{{\left(\Delta^{2}b^{2}\xi +\beta^{2}C_{L}-4b\xi C_{L}\right)}^{2}}$$

By calculation, we know that $\pi_{m}^{d*}>\pi_{r}^{d*}$. This indicates that in a manufacturer-led closed-loop supply chain, depending on the wholesale price of the manufacturer, if the retailer’s adjustment space is small, the profits will be lower than those of the manufacturer.

#### 3.3.2. Optimal Decisions with CSR under Centralized Decision Making

Under centralized decision making, the manufacturer and the retailer make their corresponding decisions for the purpose of maximizing of the system profits. In this case, the objective profit function for the whole supply chain is as follows:

(26) $$\pi_{c}=[a-bp+\beta e_{m}](p-c_{m}+\Delta \tau )-\frac{1}{2}\xi e_{m}{}^{2}-\frac{1}{2}C_{L}\tau^{2}$$

**Theorem 4.**  *In the supply chain with CSR, let* $p^{c*},\tau^{c*},e_{m}{}^{c*}$ *represent the optimal retailer channel price, the optimal manufacturer recovery rate, and the corporate social responsibility level, respectively, under centralized decision making. If the parameter* $C_{L}\geq \frac{b\Delta \xi (a+b\Delta -bc_{m})}{2b\xi -2\beta^{2}}$ *,* 0<τ<1 *, and there exists a unique value to maximize the profits of the supply chain, the following will hold true:*

(27) $$p_{}^{c*}=\frac{\Delta^{2}ab\xi -b\xi C_{L}c_{m}+\beta^{2}C_{L}c_{m}-a\xi C_{L}}{\Delta^{2}b^{2}\xi -2b\xi C_{L}+\beta^{2}C_{L}}$$

(28) $$\tau_{}^{c*}=-\frac{\Delta b\xi (a-bc_{m})}{\Delta^{2}b^{2}\xi -2b\xi C_{L}+\beta^{2}C_{L}}$$

(29) $$e_{m}{}_{}^{c*}=-\frac{\beta C_{L}(a-bc_{m})}{\Delta^{2}b^{2}\xi -2b\xi C_{L}+\beta^{2}C_{L}}$$

The proof is shown in Appendix A.

According to Theorem 4, in the supply chain with CSR, by putting the above optimal solutions into the demand function under centralized decision making, we can determine the optimal demand as follows:

(30) $$D^{c*}=-\frac{b\xi C_{L}(a-bc_{m})}{\Delta^{2}b^{2}\xi -2b\xi C_{L}+\beta^{2}C_{L}}$$

Then, the profit function of the whole closed-loop supply chain can be obtained as follows:

(31) $$\pi^{c*}=-\frac{1}{2}\frac{\xi C_{L}{\left(a-bc_{m}\right)}^{2}}{\left[(-2b\xi +\beta^{2})C_{L}+\Delta^{2}b^{2}\xi \right]}$$

#### 3.3.3. Comparative Analysis under the Different Decision Situations

**Theorem 5.**  *Let* $\Delta_{\tau }^{c}$ *stand for the difference in the manufacturer’s optimal recovery rate from the closed-loop supply chain with and without CSR, the value of which can be calculated as follows:*

(32) $$\Delta_{\tau }^{c}=\tau^{c*}-\tau_{N}^{c*}=\frac{\Delta (a-bc_{m})\beta^{2}C_{L}}{\left(\Delta^{2}b^{2}\xi -2b\xi C_{L}+\beta^{2}C_{L})(\Delta^{2}b-2C_{L}\right)}>0$$

The proof is shown in Appendix A.

It is clear that with the introduction of CSR in the closed-loop supply chain, the manufacturer’s product recovery rate is higher than that without CSR. It benefits from the improvement of awareness of resource saving and environmental protection throughout the whole closed-loop supply chain by supply chain members, which causes manufacturers to pay more attention to product recycling.

**Theorem 6.**  *Let* $\Delta_{p}^{c}$ *stand for the difference in the retailer’s optimal price between cases with and without the introduction of CSR in the closed-loop supply chain. Through calculation, we can obtain:*

(33) $$\Delta_{p}^{c}=p^{c*}-p_{N}^{c*}=-\frac{(\Delta^{2}b-C_{L})(a-bc_{m})\beta^{2}C_{L}}{(\Delta^{2}b^{2}\xi -2b\xi C_{L}+\beta^{2}C_{L})(\Delta^{2}b-2C_{L})b}>0$$

The proof is shown in Appendix A.

According to Theorem 6, it can be determined that the retail price of the product with CSR is higher than that without CSR. The reason for this is that the consideration of CSR inevitably entails corresponding costs, and the majority of enterprises will share these increased costs via other mechanisms, such as sales prices. Therefore, the question of whether to invest and how much to invest in CSR is of reference and guiding significance in enterprise management.

**Theorem 7.**  *In the closed-loop supply chain, let* $\Delta_{\pi }^{c},\Delta_{D}^{c}$ *represent the difference in overall profits and sales volume, respectively, of the supply chain between cases with and without the introduction of CSR in the closed-loop supply chain, the values of which can be determined as follows:*

(34) $$\Delta_{\pi }^{c}=\frac{1}{2}\frac{{\left(a-bc_{m}\right)}^{2}\beta^{2}C_{L}{}^{2}}{(\Delta^{2}b^{2}\xi -2b\xi C_{L}+\beta^{2}C_{L})(\Delta^{2}b-2C_{L})b}>0$$

(35) $$\Delta_{D}^{c}=\frac{(a-bc_{m})\beta^{2}C_{L}{}^{2}}{\left(\Delta^{2}b^{2}\xi -2b\xi C_{L}+\beta^{2}C_{L})(\Delta^{2}b-2C_{L}\right)}>0$$

The proof is shown in Appendix A.

According to Theorem 7, when upstream and downstream enterprises make centralized decisions based on overall profits, corporate social responsibility plays a positive role in the overall profits and sales volume of the supply chain. For the closed-loop supply chain, CSR can not only optimize and improve the structural adjustment of upstream and downstream product production, sales, and recycling, it can also promote the supervision of CSR undertaken by society as a whole, which will be more conducive to the development and operation of the social economy.

**Theorem 8.**  *In the closed-loop supply chain, let* $\Delta_{\tau }^{d},\Delta_{w}^{d},\Delta \pi_{m}^{d}$ *represent the difference between the manufacturer’s optimal recovery rate, wholesale price, and profits, respectively, between cases with and without the introduction of CSR in the closed-loop supply chain. Then, their values can be determined as follows:*

(36) $$\Delta_{\tau }^{d}=\frac{\Delta (a-bc_{m})\beta^{2}C_{L}}{\left(\Delta^{2}b^{2}\xi -4b\xi C_{L}+\beta^{2}C_{L})(\Delta^{2}b-4C_{L}\right)}>0$$

(37) $$\Delta_{{}_{w}}^{d}=-\frac{\beta^{2}C_{L}(\Delta^{2}b-2C_{L})(-bc_{m}+a)}{(\Delta^{2}b^{2}\xi -4b\xi C_{L}+\beta^{2}C_{L})(\Delta^{2}b-4C_{L})b}>0$$

(38) $$\Delta \pi_{m}^{d}=\frac{1}{2}\frac{{\left(a-bc_{m}\right)}^{2}\beta^{2}C_{L}{}^{2}}{(\Delta^{2}b^{2}\xi -4b\xi C_{L}+\beta^{2}C_{L})(\Delta^{2}b-4C_{L})b}>0$$

The proof is shown in Appendix A.

According to Theorem 8, we can see that in the manufacturer-led closed-loop supply chain, the manufacturer’s product recovery rate is higher with CSR than that without CSR. This is because the introduction of CSR enhances awareness of green environmental protection, and enterprises carry out the disposal and treatment of waste products in a green and scientifically sound manner, which plays an important role in resource conservation and protection and affects the operation and production of enterprises directly or indirectly. Therefore, the profits of the manufacturer with CSR input are higher than those without CSR input. Correspondingly, CSR will surely increase the production and operation costs of the manufacturer. In order to preserve their own profits, the manufacturer will raise their wholesale prices to adjust. Therefore, wholesale prices after the introduction of CSR will be much higher than those without its introduction.

**Theorem 9.**  *In the closed-loop supply chain, let* $\Delta_{p}^{d},\Delta \pi_{r}^{d},\Delta_{{}_{D}}^{d}$ *represent the difference in the retailer’s optimal sales price, profits, and product sales volume, respectively, between cases with and without the introduction of CSR in the closed-loop supply chain, which can be determined as follows:*

(39) $$\Delta_{{}_{p}}^{d}=-\frac{(\Delta^{2}b-3C_{L})(a-bc_{m})\beta^{2}C_{L}}{(\Delta^{2}b^{2}\xi -4b\xi C_{L}+\beta^{2}C_{L})(\Delta^{2}b-4C_{L})b}>0$$

(40) $$\Delta \pi_{r}^{d}=-\frac{2(\Delta^{2}b-3C_{L})(\Delta^{2}b^{2}\xi -4b\xi C_{L}+\frac{1}{2}\beta^{2}C_{L}){\left(a-bc_{m}\right)}^{2}\beta^{2}C_{L}{}^{3}}{{\left(\Delta^{2}b^{2}\xi -4b\xi C_{L}+\beta^{2}C_{L}\right)}^{2}{\left(\Delta^{2}b-4C_{L}\right)}^{2}b}>0$$

(41) $$\Delta_{{}_{D}}^{d}=\frac{(a-bc_{m})\beta^{2}C_{L}{}^{2}}{\left(\Delta^{2}b^{2}\xi -4b\xi C_{L}+\beta^{2}C_{L})(\Delta^{2}b-4C_{L}\right)}>0$$

The proof is shown in Appendix A.

According to Theorem 9, when introducing CSR into the closed-loop supply chain, the sales prices of the retailer’s products will be higher than those without the introduction of CSR. On the one hand, the manufacturer increases the wholesale price. On the other hand, the input of CSR improves the quality and assignment of the whole process of production, transportation, sales, and recycling. Similarly, the investment of CSR has a positive impact on the reputation of enterprises and the popularity of products. The sales volume of products after introducing CSR will obviously be higher than that without introducing CSR, and the sales price of products is higher, so the profits of the retailer will obviously be higher than those without introducing CSR.

**Theorem 10.**  *In the manufacturer-led closed-loop supply chain with CSR, when*$2bC_{L}-\Delta^{2}b^{2}>0$*and*$\frac{\Delta^{2}b^{2}\xi +C_{L}\beta^{2}}{4}-b\xi C_{L}<0$*, the product sales price*p*will decrease with increasing CSR cost coefficient*ξ. (*The proof is shown in*Appendix A*).*

**Theorem 11.**  *In the manufacturer-led closed-loop supply chain with CSR, when*$2bC_{L}-\Delta^{2}b^{2}>0$*and*$\frac{\Delta^{2}b^{2}\xi +C_{L}\beta^{2}}{4}-b\xi C_{L}<0$*, the manufacturer’s product recovery rate*τ*and wholesale price*w*will decrease with increasing CSR cost coefficient*ξ. (*The proof is shown in*Appendix A*).*

**Theorem 12.**  *In the manufacturer-led closed-loop supply chain with CSR, when*$2bC_{L}-\Delta^{2}b^{2}>0$*and*$\frac{\Delta^{2}b^{2}\xi +C_{L}\beta^{2}}{4}-b\xi C_{L}<0$*, the manufacturer’s CSR*$e_{m}$*will decrease with increasing CSR cost coefficient*ξ. (*The proof is shown in*Appendix A*).*

**Theorem 13.**  *In the manufacturer-led closed-loop supply chain with CSR, when*$2bC_{L}-\Delta^{2}b^{2}>0$*and*$\frac{\Delta^{2}b^{2}\xi +C_{L}\beta^{2}}{4}-b\xi C_{L}<0$*, the profits of the manufacturer, retailer and supply chain will all decrease with increasing CSR cost coefficient*ξ. (*The proof is shown in*Appendix A*).*

### 3.4. A Two-Part Tariff Coordination Mechanism Considering CSR

On the basis of the above analysis, various decisions and overall profits of the closed-loop supply chain under centralized decision making are better than those under decentralized decision making. However, some supply chain members often face loss, and then the supply chain produces conflicts. In this manufacturer-led closed-loop supply chain, the manufacturer often takes some measures, such as the provision of subsidies, in order to stimulate retailers to undertake CSR and improve the level and efficiency of supply chain operation. Based on the relevant literature (Zhao Hai-xia [38], Ai et al. [39]), from the angle the of the wholesale price negotiation and subsidy, in this section, we will propose a two-step pricing contract mechanism. The basic idea is as follows: Firstly, the manufacturer first sells w below the market price to the retailer, and the retailer provides the manufacturer with a fixed fee F as an exchange, so as to encourage upstream and downstream partners to take the optimal solution under centralized decision making. In the following, TPT represents a two-part tariff mechanism, and MS represents manufacturer-led. Therefore, under the two-part tariff contract ($w_{{}^{MS}}^{TPT}$, $F_{MS}^{TPT}$), the manufacturer’s decision problem can be expressed as follows:

(42) $${\underset{\left(MS\right)}{\pi }}_{{}_{m}}^{TPT}=(w_{{}^{MS}}^{TPT}-c_{m}+\Delta \tau )(a-bp+\beta e_{m})-\frac{1}{2}\xi e_{m}{}^{2}-\frac{1}{2}C_{L}\tau^{2}+F_{MS}^{TPT}$$

(43) $${\underset{\left(MS\right)}{\pi }}_{r}^{TPT}=(p-w)(a-bp+\beta e_{m})-F_{MS}^{TPT}$$

There is also a constraint ${\underset{\left(MS\right)}{\pi }}_{r}^{TPT}=(p-w)(a-bp+\beta e_{m})-F\geq \pi_{r}{}^{d*}(MS)$ in the above model indicating that both parties are willing to participate if they can at least obtain the retained profits under decentralized decision making.

Conclusion 1. *Let* $w_{MS}^{TPT},F_{MS}^{TPT}$ *represent the wholesale price and the fixed fee provided by the retailer to the manufacturer, respectively, under the two-part tariff contract; therefore, we can obtain:*

(44) $$w_{MS}^{TPT}=\frac{1}{4}\frac{\left(-8\xi^{2}b^{2}c_{m}+2\beta^{4}c_{m}-4\xi a\beta^{2}\right)C_{L}^{2}+4\Delta^{2}b\left(\xi \left(\frac{1}{2}\beta^{2}c_{m}+\xi a\right)b-\frac{1}{4}\beta^{4}c_{m}+\frac{1}{2}a\xi \beta^{2}\right)C_{L}-\Delta^{4}\xi ab^{2}\beta^{2}}{\left(\Delta^{2}b-2C_{L}\right)\left(\left(\xi^{2}b^{2}+\frac{1}{2}\xi b\beta^{2}-\frac{1}{4}\beta^{4}\right)C_{L}-\frac{1}{4}\Delta^{2}\xi b^{2}\beta^{2}\right)}$$

(45) $$F_{MS}^{TPT}=\frac{4C_{L}{}^{2}{\left((a-bw_{{}^{MS}}^{TPT})\xi +\frac{1}{2}(w_{{}^{MS}}^{TPT}-c_{m})\beta^{2}\right)}^{2}}{b{\left(4\xi C_{L}-\Delta^{2}\beta^{2}\right)}^{2}}-\pi_{r}{}^{d*}(MS)$$

## 4. Numerical Experiment

In recent years, with increased attention being paid to the concept of green environmental protection and sustainable development, an increasing number of enterprises have begun to focus on the construction of CSR, but meanwhile, they do not know how to manage and implement it. To show the influence of the introduction of CSR on the decision-making process of each member of a closed-loop supply chain with the manufacturer as the channel leader and the usefulness of the coordination mechanism, this chapter presents our conclusions by means of an example. In this section, we will take the closed-loop supply chain formed by a manufacturer (enterprise A) and its cooperative retailer (enterprise B) as an example. Enterprise A is the only recycling party, and the whole system runs as stated in the previous model. First, the products of enterprise A are sold wholesale to downstream enterprise B, and then enterprise B pushes the products to the market terminal. In the recycling process, enterprise A recycles from the sales market or buyers.

Firstly, on the basis of the decision-making model of the manufacturer-led closed-loop supply chain considering CSR proposed above, the influence of the change in each decision variable on the income, consumer market demand and product price of upstream and downstream enterprises is clearly and intuitively shown by graphs, and then the optimal decision-making product is solved for A and B. According to the actual situation and the difficulty of the calculation, considering the decision-making model in which the manufacturer is the recycler and is investing in CSR, combined with the relevant research of Ma et al. [40] and Song et al. [41], the initial experimental data are selected as follows after certain processing: $a=100,c_{m}=5,\Delta =2$, β=8, b=10. In addition, for comparative analysis, $C_{L}$ and ξ are taken as variable values, respectively; that is, when $C_{L}$ is a variable, ξ=30. $C_{L}=150$ when ξ is a variable. As a variable, the value of $C_{L}$ is between [80,150] and that of ξ is between [7, 30]; all parameters in this interval satisfy $C_{L}\beta^{2}+\Delta^{2}b^{2}\xi -2b\xi C_{L}<0$, $\frac{\Delta^{2}b^{2}\xi +C_{L}\beta^{2}}{4}-b\xi C_{L}<0$, $2bC_{L}-\Delta^{2}b^{2}>0$ and $C_{L}>\frac{\Delta b\xi (a-bc_{m}+\Delta b)}{2b\xi -\beta^{2}}$, so it has practical significance.

### 4.1. The Influence of the Recovery Scale Parameter on Decision Variables under Different Decision Situations

**Figure 1:**
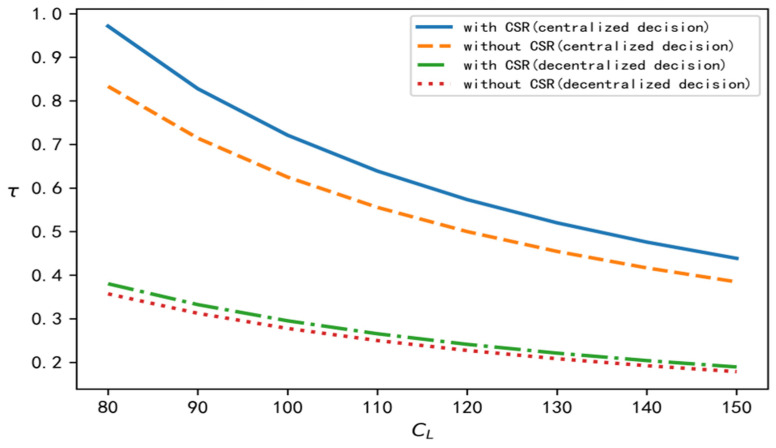
Change trend of product recovery rate.

**Figure 2:**
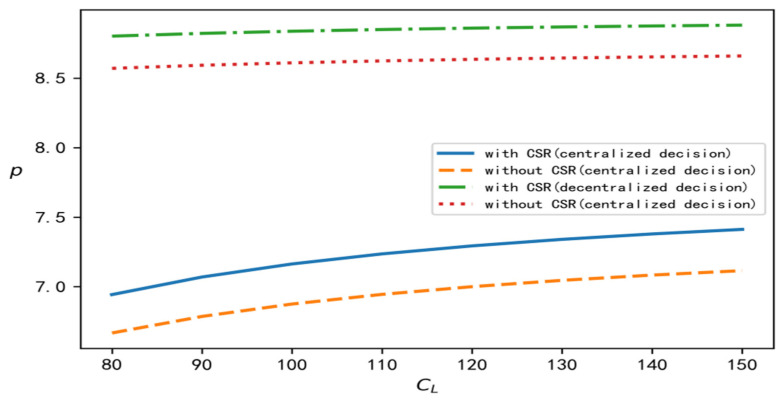
Change trend of product price.

**Figure 3:**
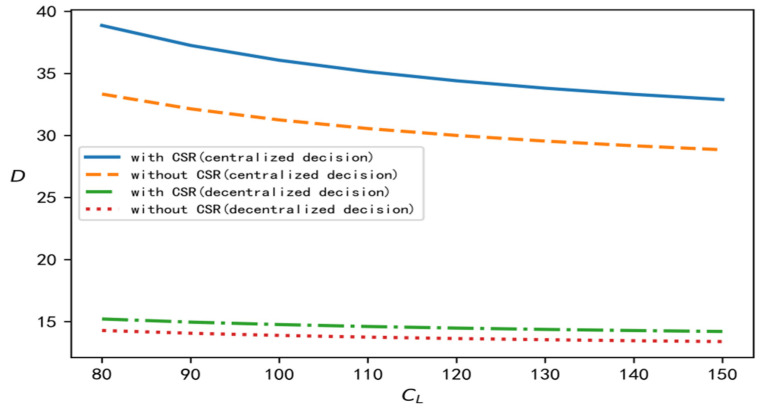
Change trend of demand.

**Figure 4:**
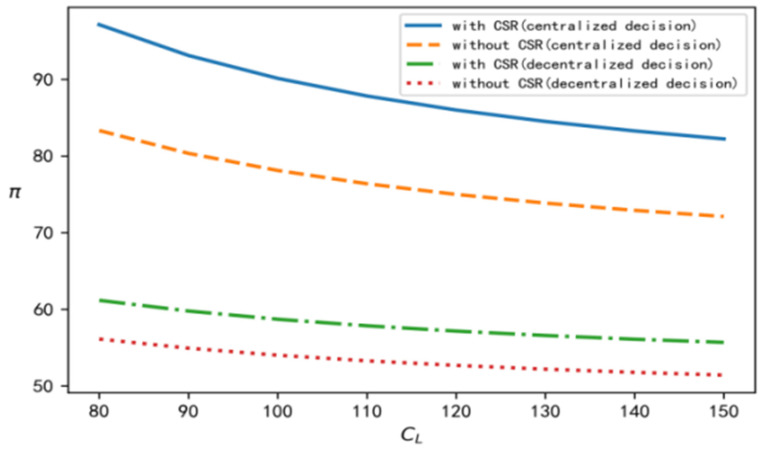
Change trend of the overall profits of closed-loop supply chain.

**Figure 5:**
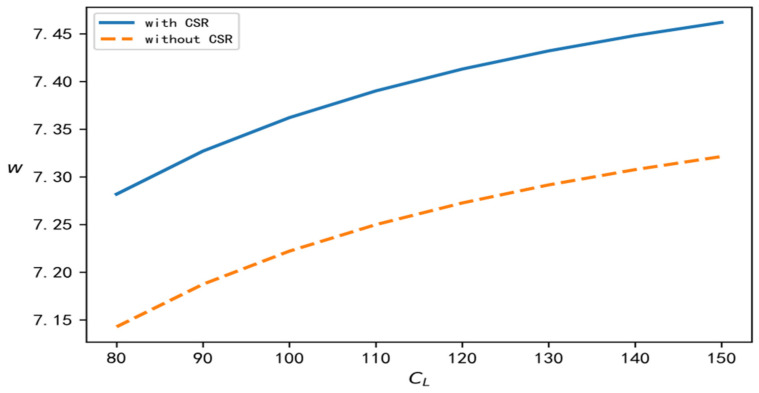
Change trend of the wholesale price.

According to the above analysis process and conclusion, in order to perform a better comparison, centralized, decentralized, and with and without CSR are selected at the same time, and relevant parameters are brought into the software for simulation calculation and analysis. In this section, we discuss the influence of the recovery scale parameter on decision variables such as product recovery rate, product price, demand, overall profits of the supply chain, and the wholesale price under different decision situations. To improve the comparative analysis, $C_{L}$ is taken to be an independent variable. On the one hand, $C_{L}$ is involved in all models, and on the other hand, it reflects the recovery cost of the closed-loop supply chain. According to the models established in this paper and the above data, the change trend of each variable with changes in the value of $C_{L}$ can be determined under four different decision situations, as shown in Figure 1, Figure 2, Figure 3, Figure 4 and Figure 5, respectively.

Figure 1, Figure 2, Figure 3, Figure 4 and Figure 5 show the changes in market demand, the wholesale price of products, the income of upstream and downstream members, and product recovery in the manufacturer-led closed-loop supply chain. It can be observed that whatever the values of recovery rate, market demand, member income, or sales price and wholesale price of products are, when the upstream and downstream enterprises cooperate for common profits, their level is at its best, thus realizing the objective of maximizing their respective profits, indicating that more efficient results can be derived in this way. In addition, in terms of recovery rate, product pricing, market demand, overall income of the closed-loop supply chain, and wholesale price of products, when the factor of CSR is added, their respective levels change significantly, and are better than their levels without CSR. The reasons for this may be as follows: CSR increases the product recovery rate, reprocesses underused products, saves resources, optimizes product packaging, makes the production process green, and protects labour rights and profits, which, to a certain extent, enhances the enterprise’s product quality, enhances the reputation of the enterprises, and promotes the popularity of products among consumers.

### 4.2. The Influence of CSR Cost Coefficient on Decision Variables

**Figure 6:**
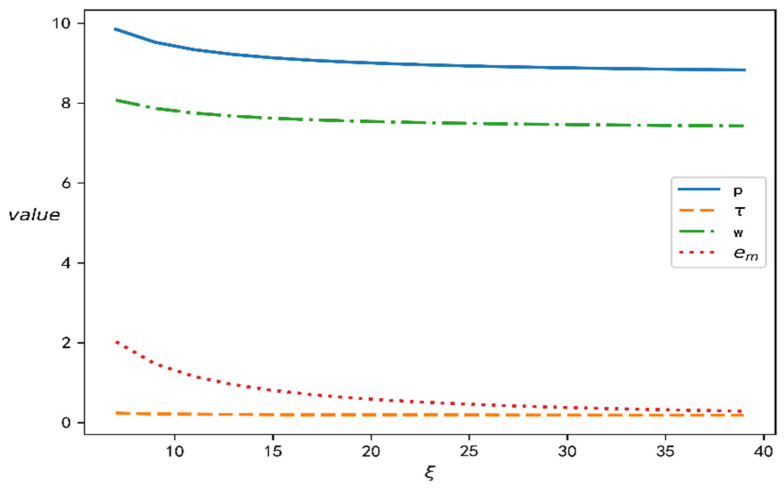
The influence of the parameter ξ on decision variables of closed-loop supply chains.

According to the models established in this paper and the above data, we can determine the influence of the parameter ξ on the decision variables of the closed-loop supply chain, as shown in Figure 6. In Figure 6, the manner in which the increasing cost affects the upstream and downstream enterprises and how it changes after the introduction CSR can be observed. First of all, when the cost ξ increases, the level of CSR efforts decrease, which means that the greater the amount of funds required, the lower the willingness of an enterprise to fulfil CSR. Secondly, the product recovery rate does not change fundamentally, which shows that there is no strong correlation between CSR and product recovery rate in the closed-loop supply chain. In reality, recycling waste products is carried out to pursue the maximization of benefits, so it is not affected by the strength of CSR. Finally, the wholesale price and the retail price of the products first slowly decrease, and then they gradually stop changing. This provides a good inspiration for enterprise managers, in that they should see long-term advantages and disadvantages in the process of undertaking corporate social responsibility. The addition of CSR improves the efficiency of enterprises and promotes the market demand, while the price remains in a reasonable range, demonstrating a positive effect on the whole. The disadvantage lies in the fact that if the input required for CSR continues to increase, this reduces income and breaks the equilibrium of the development of upstream and downstream enterprises. Therefore, CSR input should remain within a suitable range.

### 4.3. Two-Part Tariff Coordination Result Analysis

Next, we further analyse and verify the impact of changes in CSR input on the decision-making process of each member in the closed-loop supply chain and the usefulness of the two-part tariff contract mechanism proposed above. To ensure comparability and consistency, we consider a decision model in which the manufacturer is the recycler and CSR investor, and also consider the practical significance of this model in parameter setting. On the basis of the relevant literature, the initial experimental data were selected as follows: $a=100,c_{m}=5,\Delta =2,C_{L}=150$. Secondly, the range of ξ was [6,60]. According to the established coordination mechanism, by computation, we obtained the coordination results presented in Table 2. On the basis of the calculation results, it can be seen that the recovery rate increases before and after coordination, and the level of CSR efforts also improves. However, with increasing cost, $e_{m}$ gradually shows a decreasing trend, which is also in line with reality. On the other hand, the wholesale price and the retail price of products after coordination are optimized, and are lower than those before coordination, with the price reduction inevitably promoting increased product demand, resulting in an overall growth in profits. In addition, both retail price and wholesale price decrease with increasing ξ. On the whole, the coordinated closed-loop supply chain achieves its previous goals, and the wholesale price, retail price, product recovery rate and CSR level are optimized compared with the previous ones. For consumers, this means that they are able to buy more affordable and valuable products. For enterprises, this means that they can achieve a double harvest in terms of both product quality and corporate profits.

**Figure 7:**
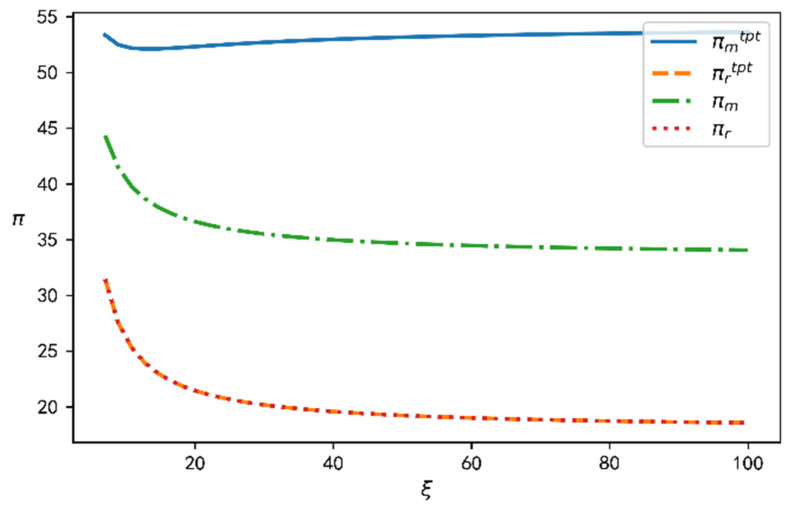
Change in profits before and after coordination.

According to the coordination mechanism established in this paper and the above data, the profit changes can be determined for the supply chain, the manufacturer, and the retailer before and after coordination with respect to CSR cost coefficient, as shown in Figure 7. According to Figure 7, the profits of the manufacturer after coordination are much higher than those before. At the same time, it can be seen that the retailer’s profits are the same before and after coordination, because the retailer’s profits are no lower than their profits under decentralized decision making.

In summary, the two-part tariff coordination model designed in this paper can effectively deal with the problem of profit loss under decentralized decision making in a closed-loop supply chain dominated by the manufacturer, which also provides ideas for the subsequent development of enterprises.

## 5. Management Implications

(1) From the perspective of the manufacturer, this analysis shows that the continuous increase in CSR cost will lead to a decrease in enterprise revenue and an increase in product price, which must be maintained within the appropriate range to achieve the maximum effect. On this basis, enterprises can amplify the positive impact of CSR through reasonable publicity and marketing means, and give full play to the effect of CSR.
(2) From the perspective of the retailer, the analysis shows that the manufacturer’s CSR level directly affects the retailer’s decision. If the manufacturer has negative CSR problems, the retailer’s profit and reputation will deteriorate greatly. Therefore, retailers should incorporate CSR into their assessment indicators when selecting partners. In addition, in the closed-loop supply chain, when the manufacturer invests in CSR, the retailer’s selling price and sales volume will increase. However, this could turn off price-oriented consumers, at the cost of a portion of sales volume for the retailer. Therefore, the retailer should set an appropriate selling price in order to increase their final profit.
(3) For the whole supply chain, CSR input intensifies the supply chain conflict. The manufacturer’s input into CSR increases the sales volume of the retailer, which, to some extent, affects the enthusiasm of the manufacturer. Although the manufacturer increases their product recycling rate due to the influence of CSR, the effect is not ideal. Therefore, a coordination mechanism is still needed to promote the enthusiasm of all parties to promote the healthy development of the supply chain.

## 6. Conclusions

In this paper, we explored the optimal decisions of supply chains with and without CSR under centralized and decentralized decision making and the influence of CSR on manufacturer-led closed-loop supply chain, and established a two-part tariff coordination mechanism. According to the analysis of the models, some conclusions could be drawn as follows:

(1) When upstream and downstream enterprises cooperate for common profits, the optimal decisions of the supply chain members under centralized decision making are better than those under decentralized decision making, and are best able to meet the objective of maximizing their own profits.
(2) When introducing CSR into the closed-loop supply chain, the level of recovery rate, product pricing, market demand, the overall profits of the closed-loop supply chain and the wholesale price of the products are all better than those without the introduction of CSR, and CSR has a positive effect. The increase in the input cost of CSR will reduce the level of CSR, as well as the wholesale price, product recovery rate, and retail price of the products.
(3) The two-part tariff contract can effectively alleviate the conflicts resulting from different decisions between upstream and downstream enterprises and supply chain profits loss, thus improving the profits of the manufacturer, the retailer, and the whole supply chain, meaning that decision-making processes reach or approach their best level under centralized decision making.

Although the validity of the optimal decisions and the effectiveness of the coordination mechanism was confirmed, our research still has some limitations. Our supply chain channel leader was the manufacturer, not the retailer, who is closer to the consumer. The product recycling party was assumed to be the manufacturer, regardless of the retailer or third party. In addition, in order to facilitate calculation, it was assumed that there was consistency between recycled products and new products, and no price difference between them. However, in reality this is often not the case. These are also required for further strengthening and improvement in the later period.

## Figures and Tables

**Table 1:** Parameter description of the model.

Parameter	Description
ω	The wholesale price of the manufacturer
P	The retail price of the product
D(D=a−bp)	The market demand function
a(a>0)	Market size
b(b>0)	Price sensitivity coefficient
$C_{L}(C_{L}>0)$	Recovery Scale parameter
ξ(ξ>0)	CSR cost coefficient
β(β>0)	Sensitivity coefficient
$c_{m}$	The unit cost of the new product
$c_{r}$	The unit cost of the re-manufactured product
τ(0≤τ≤1)	Recovery rate of waste products
Δ	Average production cost of manufacturers
C(τ)	Recovery cost
$e_{{}_{m}}$	Level of corporate social responsibility
$\pi_{{}_{m}}$	The profits of the manufacturer
$\pi_{r}$	The profits of the retailer
π	The profits of the supply chain
N	Without thinking about CSR
$d^{{}^{*}}$	Optimal decision under decentralized decision making
$c^{*}$	Optimal decision under centralized decision making
d	In the case of decentralized decision making
c	In the case of centralized decision making
$\Delta_{p}^{c}$	The retailer’s optimal price difference when CSR is introduced or not introduced
$\Delta_{\pi }^{c}$	The overall profits of the supply chain difference when CSR is introduced or not introduced
$\Delta_{D}^{c}$	The sales volume difference when CSR is introduced or not introduced

**Table 2:** The results before and after coordination.

ξ	τ	$e_{m}$	p	w	$\pi_{m}$	$\pi_{r}$	$\tau^{TPT}$	$e_{m}{}^{TPT}$	$p^{TPT}$	$w^{TPT}$	$\pi_{m}{}^{TPT}$	$\pi_{r}{}^{TPT}$
6	0.250	0.050	10.125	8.250	46.875	35.156	0.303	1.509	8.933	6.657	54.376	35.156
10	0.216	0.026	9.418	7.802	40.409	26.127	0.313	0.545	8.085	5.735	52.274	26.127
20	0.195	0.026	9.003	7.539	36.621	21.458	0.338	0.144	7.579	5.042	52.316	21.458
30	0.189	0.008	8.883	7.462	35.511	20.177	0.351	0.066	7.422	4.792	52.697	20.177
40	0.187	0.006	8.825	7.425	34.981	19.579	0.358	0.038	7.345	4.660	52.970	19.579
50	0.185	0.004	8.791	7.404	34.671	19.233	0.363	0.024	7.299	4.579	53.163	19.233
60	0.184	0.004	8.768	7.390	34.467	19.007	0.366	0.017	7.269	4.524	53.304	19.007

## Data Availability

Requests for the data used to support the findings of this study will be considered by the corresponding author (email: clly1985528@163.com).

## Appendix A

**Proof of Theorem 1.** According to Equations (4) and (5) and backward induction, by taking the derivative of retailer’s profit function over the price p and letting it be equal to 0, that is $\frac{\partial \pi }{\partial p}=-bp+a-(p-w)b=0$, we can obtain the following expression:

(A1)
$$p=\frac{1}{2}\frac{bw+a}{b}$$

By taking the derivative of the retailer’s profit function over the price, we can determine $\frac{\partial^{2}\pi }{\partial^{2}p}=-2b<0$; thus, there exists an optimal value for maximizing the retailer’s profits.

By substituting Equation (A1) into Equation (5), and calculating the derivation with respect to w and τ, we can obtain the following expressions:

(A2) $$\frac{\partial \pi }{\partial w}=-bw-\frac{1}{2}\Delta b\tau +\frac{1}{2}bc_{m}+\frac{1}{2}a=0$$

(A3) $$\frac{\partial \pi }{\partial \tau }=-\frac{1}{2}\Delta bw+\frac{1}{2}\Delta a-C_{L}\tau =0$$

Through the second derivation, we can construct the Hessian matrix:

(A4) $$H_{3.3.2}= [\begin{array}{cc} -b & -\frac{1}{2}\Delta b\\ -\frac{1}{2}\Delta b & -C_{L} \end{array}]=bC_{L}-\frac{1}{4}\Delta^{2}b^{2}$$

If and only if $bC_{L}-\frac{1}{4}\Delta^{2}b^{2}>0$, $\pi_{m}^{N}$ is a joint concave function about w and τ, and it has optimal values $w_{N}^{{}^{d*}}$ and $\tau_{N}^{{}^{d*}}$, which can be determined as a negative definite matrix. Then, its stable point ($w_{N}^{{}^{d*}},\tau_{N}^{{}^{d*}}$) is the maximum point of $\pi_{m}$, that is, the optimal solution of function $\pi_{m}^{N}$. Therefore, by setting the first derivative to be equal to zero, we can obtain the results of Theorem 1. □

**Proof of Theorem 2.** By deriving Equation (6) with respect to p and t, we can set them to be zero as shown below:

(A5)
$$\frac{\partial \pi^{N}}{\partial p}=-bp+a-(\Delta \tau +p-c_{m})b=0$$

(A6)
$$\frac{\partial \pi^{N}}{\partial \tau }=\Delta (a-bp)-C_{L}\tau =0$$

According to Equation (10), we can obtain the stable point $\left(p,\tau )=(\frac{\Delta^{2}ab-bC_{L}c_{m}-aC_{L}}{b(\Delta^{2}b-2C_{L})},-\frac{\Delta (a-bc_{m})}{\Delta^{2}b-2C_{L}}\right)$. Then, by calculating the second derivative function of Equation (6) with respect to p and t, we can determine the Hessian matrix of supply chain profit function $H_{3.3.1}= [\begin{array}{cc} -2b & -\Delta b\\ -\Delta b & -C_{L} \end{array}]$. If and only if $2bC_{L}-\Delta^{2}b^{2}>0$, that is $C_{L}>\frac{\Delta^{2}b}{2}$, then it is a negative matrix, and so the stable point ($p_{N}^{c*},\tau_{N}^{c*}$) is the maximum point, which is the optimal solution of the total profits of the closed-loop supply chain. □

**Proof of Theorem 3.** By taking the second derivative of Equation (17), we can get $\frac{\partial^{2}\pi_{{}_{r}}^{d}}{\partial p^{2}}=-2b<0$, so $\pi_{r}^{d}$ is a concave function of p, and there exists an optimal value $p_{}^{d*}$. If the first derivative is 0, the reaction function can be obtained:

(A7)
$$p=\frac{bw+\beta e_{m}+a}{2b}$$

Then, we can determine the Hessian matrix for the manufacturer as follows:

(A8) $$H(w,e_{m},\tau )=\left|\begin{array}{ccc} \frac{\partial^{2}\pi_{m}^{d}}{\partial w^{2}} & \frac{\partial^{2}\pi_{m}^{d}}{\partial w\partial e_{m}} & \frac{\partial^{2}\pi_{m}^{d}}{\partial w\partial \tau }\\ \frac{\partial^{2}\pi_{m}^{d}}{\partial e_{m}\partial w} & \frac{\partial^{2}\pi_{m}^{d}}{\partial e_{m}{}^{2}} & \frac{\partial^{2}\pi_{m}^{d}}{\partial e_{m}\partial \tau }\\ \frac{\partial^{2}\pi_{m}^{d}}{\partial \tau \partial w} & \frac{\partial^{2}\pi_{m}^{d}}{\partial \tau \partial e_{m}} & \frac{\partial^{2}\pi_{m}^{d}}{\partial \tau^{2}} \end{array}\right|=\left|\begin{array}{ccc} -b & \frac{\beta }{2} & -\frac{\Delta b}{2}\\ \frac{\beta }{2} & -\xi & \frac{\Delta \beta }{2}\\ -\frac{b\Delta }{2} & \frac{\Delta \beta }{2} & -C_{L} \end{array}\right|=\frac{\Delta^{2}b^{2}\xi +C_{L}\beta^{2}}{4}-b\xi C_{L}$$

According to Equation (A5), because $b\xi -\frac{\beta^{2}}{4}>0$, then $\frac{\Delta^{2}b^{2}\xi +C_{L}\beta^{2}}{4}-b\xi C_{L}<0$, so: $C_{L}>\frac{\Delta^{2}b^{2}\xi }{4b\xi -\beta^{2}}$. Therefore, it can be verified that $\pi_{{}_{m}}^{d}$ is the joint concave function of $w,\tau ,e_{m}$. According to the first derivative, we can obtain $\tau^{d^{*}},w^{d^{*}},e_{m}{}^{d^{*}}$ as the result of Theorem 3. 0<τ<1, so $0<\tau^{d*}=-\frac{\Delta b\xi (a-bc_{m})}{\Delta^{2}b^{2}\xi -4b\xi C_{L}+\beta^{2}C_{L}}<1$, then $C_{L}>\frac{\Delta b\xi (a-bc_{m}+\Delta b)}{2b\xi -\beta^{2}}$, so $C_{L}>\max\{\frac{\Delta b\xi (\Delta b+a-bc_{m})}{2b\xi -\beta^{2}},\frac{\Delta b\xi (\Delta b)}{4b\xi -\beta^{2}}\}>\frac{\Delta b\xi (\Delta b+a-bc_{m})}{2b\xi -\beta^{2}}$. Substituting Equations (19)–(21) into $p^{d}{}^{*}$, we can obtain the value as the result of Theorem 3. □

**Proof of Theorem 4.** According to Equation (4), it is known that $\pi_{c}$ is a multivariate function of $p,e_{m},\tau$. The Hessian matrix of Equation (4) can be calculated as follows:

(A9)
$$H(p,e_{m},\tau )=\left|\begin{array}{ccc} \frac{\partial^{2}\pi_{c}}{\partial p^{2}} & \frac{\partial^{2}\pi_{c}}{\partial p\partial e_{m}} & \frac{\partial^{2}\pi_{c}}{\partial p\partial \tau }\\ \frac{\partial^{2}\pi_{c}}{\partial e_{m}\partial p} & \frac{\partial^{2}\pi_{c}}{\partial e_{m}{}^{2}} & \frac{\partial^{2}\pi_{c}}{\partial e_{m}\partial \tau }\\ \frac{\partial^{2}\pi_{c}}{\partial \tau \partial p} & \frac{\partial^{2}\pi_{c}}{\partial \tau \partial e_{m}} & \frac{\partial^{2}\pi_{c}}{\partial \tau^{2}} \end{array}\right|=\left|\begin{array}{ccc} -2b & \beta & -b\Delta \\ \beta & -\xi & \beta \Delta \\ -b\Delta & \beta \Delta & -C_{L} \end{array}\right|$$

To give each decision variable a unique value under centralized decision making, we have $\left|\frac{\partial^{2}\pi_{c}}{\partial p^{2}}\right|=-2b<0$, $\left|\begin{array}{cc} -2b & \beta \\ \beta & -\xi \end{array}\right|=2b\xi -\beta^{2}$, $\left|\begin{array}{ccc} -2b & \beta & -b\Delta \\ \beta & -\xi & \beta \Delta \\ -b\Delta & \beta \Delta & -C_{L} \end{array}\right|=C_{L}\beta^{2}+\Delta^{2}b^{2}\xi -2b\xi C_{L}$. To let the Hessian matrix be negative definite, we have $2b\xi -\beta^{2}>0$, $C_{L}\beta^{2}+\Delta^{2}b^{2}\xi -2b\xi C_{L}<0$; thus, we get: $C_{L}>\frac{\Delta^{2}b^{2}\xi }{2b\xi -\beta^{2}}$. At the same time, it has to satisfy 0<τ<1, meaning that we can obtain $0<-\frac{\Delta b\xi (a-bc_{m})}{\Delta^{2}b^{2}\xi -2b\xi C_{L}+\beta^{2}C_{L}}<1$, (because $a-bc_{m}>0$, $-\frac{\Delta b\xi (a-bc_{m})}{\Delta^{2}b^{2}\xi -2b\xi C_{L}+\beta^{2}C_{L}}>0$), and on the basis of $-\frac{\Delta b\xi (a-bc_{m})}{\Delta^{2}b^{2}\xi -2b\xi C_{L}+\beta^{2}C_{L}}<1$, we can obtain $C_{L}>\frac{\Delta b\xi (a+b\Delta -bc_{m})}{2b\xi -\beta^{2}}$. In summary, $C_{L}>\left\{\begin{array}{cc} \frac{\Delta^{2}b^{2}\xi }{2b\xi -\beta^{2}} & \frac{\Delta b\xi (a+b\Delta -bc_{m})}{2b\xi -\beta^{2}} \end{array}\right\}>\frac{\Delta b\xi (a+b\Delta -bc_{m})}{2b\xi -\beta^{2}}$. Therefore, we know that $\pi_{c}$ is a correlation concave function of $p,e_{m},\tau$, and there exist for them optimal values. Then, we calculate them separately, and take the first derivative of $\pi_{c}$ with respect to ($p,e_{m},\tau$), and make them equal to zero as follows:

(A10) $$\left\{\begin{array}{l} \frac{\partial \pi_{c}}{\partial p}=-b(p+\Delta \tau -c_{m})+[a-bp+\beta e_{m})]=0\\ \frac{\partial \pi_{c}}{\partial e_{m}}=\beta (\Delta \tau +p-c_{m})-\xi e_{m}=0\\ \frac{\partial \pi_{c}}{\partial \tau }=[a-bp+\beta e_{m}]\Delta -C_{L}\tau =0 \end{array}\right.$$

Then, we can obtain that the optimal results for each variable are those of Theorem 4. □

**Proof of Theorem 5.** According to $\Delta_{{}_{\tau }}^{c}=\tau_{}^{c*}-\tau_{N}^{c*}$, we can obtain the result. □

**Proof of Theorem 6.** Because $C_{L}>\frac{\Delta^{2}b}{2}$, there are two cases when $\Delta^{2}b-C_{L}$: (1) when $\Delta^{2}b>C_{L}>\frac{\Delta^{2}b}{2}$, (2) $C_{L}>\Delta^{2}b$. According to the above assumptions, $C_{L}>\frac{\Delta b\xi (a-bc_{m}+\Delta b)}{2b\xi -\beta^{2}}$, if $\Delta^{2}b>C_{L}>\frac{\Delta^{2}b}{2}$ is selected, it will not meet the conditions, which will destroy the conditions for obtaining the optimal value in this paper, so $C_{L}>\Delta^{2}b$. Therefore, we can obtain $\Delta_{{}_{p}}^{c}$ as the result of Theorem 6. □

**Proof of Theorem 7.** According to $\Delta_{{}_{\pi }}^{c}=\pi_{}^{c*}-\pi_{N}^{c*}$ and $\Delta_{{}_{D}}^{c}=D_{}^{c*}-D_{N}^{c*}$, we can obtain the results. □

**Proof of Theorem 8.** According to $\Delta_{\tau }=\tau^{d*}-\tau_{N}^{{}^{d*}}$, $\Delta_{{}_{w}}^{d}=w_{}^{d*}-w_{N}^{d*}$ and $\Delta \pi_{m}^{d}=\pi_{m}^{d*}-\pi_{m}^{Nd*}$, we can obtain the results. □

**Proof of Theorem 9.** According to $\Delta_{{}_{p}}^{d}=p_{}^{d*}-p_{N}^{d*}$, $\Delta \pi_{r}^{d}=\pi_{r}^{d*}-\pi_{r}^{Nd*}$ and $\Delta_{{}_{D}}^{d}=D_{}^{d*}-D_{N}^{d*}$, we can obtain the results. □

**Proof of Theorem 10.** According to Equation (20), we can see the parameters related to CSR are β and ξ, by calculating the second derivative of p with respect to β and ξ, respectively. Then, $2bC_{L}-\Delta^{2}b^{2}>0$, $\frac{\Delta^{2}b^{2}\xi +C_{L}\beta^{2}}{4}-b\xi C_{L}<0$ and $a-bc_{m}>0$, so we can obtain the following expression:

(A11)
$$\frac{\partial^{2}p^{d*}}{\partial \xi^{2}}=-\frac{2b(\Delta^{2}b-4C_{L})\beta^{2}C_{L}(\Delta^{2}b-3C_{L})(a-bc_{m})}{{\left(\Delta^{2}b^{2}\xi -4b\xi C_{L}+\beta^{2}C_{L}\right)}^{3}}>0$$

This indicates that the first-order function $\frac{\partial p^{d*}}{\partial \xi }$ is an increasing function, but $\Delta^{2}b-3C_{L}<0$, so we can obtain the following expression:

(A12) $$\frac{\partial p^{d*}}{\partial \xi }=\frac{\beta^{2}C_{L}(\Delta^{2}b-3C_{L})(a-bc_{m})}{{\left(\Delta^{2}b^{2}\xi -4b\xi C_{L}+\beta^{2}C_{L}\right)}^{2}}<0$$

Thus, the product sales price is negatively correlated with the CSR cost coefficient. □

**Proof of Theorem 11.** According to Equation (19), we calculate the first-order derivation, considering $\Delta^{2}b-2C_{L}<0$, $\Delta^{2}b-4C_{L}<0$, so we can obtain the following expression:

(A13)
$$\frac{\partial \tau^{d*}}{\partial \xi }=-\frac{\beta^{2}C_{L}\Delta b(a-bc_{m})}{{\left(\Delta^{2}b^{2}\xi -4b\xi C_{L}+\beta^{2}C_{L}\right)}^{2}}<0$$

Then, we can find that the manufacturer’s product recovery rate is negatively correlated with the cost coefficient of CSR. In the same way, Equation (26) is the reaction function of the optimal wholesale price of the manufacturer’s products, because $\Delta^{2}b-2C_{L}<0$; therefore, we can obtain the following expression:

(A14) $$\frac{\partial w^{d*}}{\partial \xi }=\frac{\beta^{2}C_{L}(\Delta^{2}b-2C_{L})(a-bc_{m})}{{\left(\Delta^{2}b^{2}\xi -4b\xi C_{L}+\beta^{2}C_{L}\right)}^{2}}<0$$

Thus, the wholesale price of manufacturer’s products is negatively correlated with the CSR cost coefficient. □

**Proof of Theorem 12.** According to Equation (21), we calculate the first-order derivation, considering $\Delta^{2}b-2C_{L}<0$, $\Delta^{2}b-4C_{L}<0$, so we can obtain the following expression:

(A15)
$$\frac{\partial e_{m}{}^{d*}}{\partial \xi }=\frac{\beta C_{L}(\Delta^{2}b-4C_{L})(a-bc_{m})}{{\left(\Delta^{2}b^{2}\xi -4b\xi C_{L}+\beta^{2}C_{L}\right)}^{2}}<0$$

Thus, the manufacturer’s CSR is negatively correlated with CSR cost coefficient. □

**Proof of Theorem 13.** According to Equations (23)–(25), we calculate the first-order derivation of these three reaction functions about the CSR cost coefficient ξ, considering $\Delta^{2}b-2C_{L}<0$, $\Delta^{2}b^{2}\xi -4b\xi C_{L}+\beta^{2}C_{L}<0$, $a-bc_{m}>0$ and all other parameters are greater than zero, so that we can obtain the following expression:

(A16)
$$\frac{\partial \pi_{m}{}^{d*}}{\partial \xi }=-\frac{1}{2}\frac{{\left(a-bc_{m}\right)}^{2}\beta^{2}C_{L}{}^{2}}{{\left(\Delta^{2}b^{2}\xi -4b\xi C_{L}+\beta^{2}C_{L}\right)}^{2}}<0$$

(A17)
$$\frac{\partial \pi_{r}{}^{d*}}{\partial \xi }=\frac{2b\xi {\left(a-bc_{m}\right)}^{2}\beta^{2}C_{L}{}^{3}}{{\left(\Delta^{2}b^{2}\xi -4b\xi C_{L}+\beta^{2}C_{L}\right)}^{3}}<0$$

(A18)
$$\frac{\partial \pi_{}{}^{d*}}{\partial \xi }=-\frac{1}{2}\frac{C_{L}{}^{2}{\left(a-bc_{m}\right)}^{2}\beta^{2}(\Delta^{2}b^{2}\xi -8b\xi C_{L}+\beta^{2}C_{L})}{{\left(\Delta^{2}b^{2}\xi -4b\xi C_{L}+\beta^{2}C_{L}\right)}^{3}}<0$$

Thus, the profits of the manufacturer, retailer and supply chain are negatively correlated with the CSR cost coefficient. □

**Proof of Conclusion 1.** According to the reverse method, by taking first-order derivation of the retailer’s profit function over to the price, we can obtain $\frac{\partial {\underset{\left(MS\right)}{\pi }}_{r}^{TPT}}{\partial p}=-bp+\beta e_{m}+a-(p-w_{MS}^{TPT})b$. Let $\frac{\partial {\underset{\left(MS\right)}{\pi }}_{r}^{TPT}}{\partial p}=0$, we can obtain $p=\frac{1}{2}\frac{bw_{MS}^{TPT}+\beta e_{m}+a}{b}$. Thus, we can obtain the following expression:

(A19)
$${\underset{\left(MS\right)}{\pi }}_{m}^{TPT}=\frac{1}{2}(a-bw_{MS}^{TPT}+\beta e_{m})w_{MS}^{TPT}-\frac{1}{2}\xi e_{m}{}^{2}-\frac{1}{2}\beta (c_{m}-\Delta \tau )e_{m}-\frac{1}{2}C_{L}\tau^{2}+\frac{1}{2}\Delta (a-bw_{MS}^{TPT})\tau +\frac{1}{2}(bw_{MS}^{TPT}-a)c_{m}+F_{MS}^{TPT}$$

Then, by taking the first derivative of $\pi_{m}$ with respect to $e_{m},\tau$, respectively, and making it 0, we can determine the following result:

(A20) $$\tau =\frac{\Delta (2bw_{MS}^{TPT}\xi -w_{MS}^{TPT}\beta^{2}+\beta^{2}c_{m}-2\xi a)}{\Delta^{2}\beta^{2}-4\xi C_{L}}$$

At this time, the retailer’s profits can be expressed as follows:

(A21) $${\underset{\left(MS\right)}{\pi }}_{{}_{r}}^{TPT}=\frac{4C_{L}{}^{2}{\left((a-bw_{MS}^{TPT})\xi +\frac{1}{2}(w_{MS}^{TPT}-c_{m})\beta^{2}\right)}^{2}}{b{\left(4\xi C_{L}-\Delta^{2}\beta^{2}\right)}^{2}}-F_{MS}^{TPT}$$

(A22) $$s.t.{\underset{\left(MS\right)}{\pi }}_{r}^{TPT}=\frac{4C_{L}{}^{2}{\left((a-bw_{MS}^{TPT})\xi +\frac{1}{2}(w_{MS}^{TPT}-c_{m})\beta^{2}\right)}^{2}}{b{\left(4\xi C_{L}-\Delta^{2}\beta^{2}\right)}^{2}}-F\geq \pi_{r}{}^{d*}(MS)$$

Under this condition, $\pi_{r}{}^{d*}(MS)=\frac{b\xi^{2}C_{L}{}^{2}{\left(a-bc_{m}\right)}^{2}}{{\left(\Delta^{2}b^{2}\xi -4b\xi C_{L}+\beta^{2}C_{L}\right)}^{2}}$. Therefore, the minimum profits should be equal to $\pi_{r}{}^{d*}(MS)$ for the retailer. It can be found that:

(A23) $$F_{MS}^{TPT}=\frac{4C_{L}{}^{2}{\left((a-bw_{MS}^{TPT})\xi +\frac{1}{2}(w_{MS}^{TPT}-c_{m})\beta^{2}\right)}^{2}}{b{\left(4\xi C_{L}-\Delta^{2}\beta^{2}\right)}^{2}}-\pi_{r}{}^{d*}(MS)$$

Based on this, by association with the manufacturer’s profit equation, we obtain:

(A24) $$\begin{array}{c} {\underset{\left(MS\right)}{\pi }}_{{}_{m}}^{TPT}=(\Delta \tau +w_{MS}^{TPT}-c_{m})(a-bp+\beta e_{m})-\frac{1}{2}\xi e_{m}{}^{2}-\frac{1}{2}C_{L}\tau^{2}\\ +\frac{4C_{L}{}^{2}{\left((a-bw_{MS}^{TPT})\xi +\frac{1}{2}(w_{MS}^{TPT}-c_{m})\beta^{2}\right)}^{2}}{b{\left(4\xi C_{L}-\Delta^{2}\beta^{2}\right)}^{2}}-(\frac{b\xi^{2}C_{L}{}^{2}{\left(a-bc_{m}\right)}^{2}}{{\left(\Delta^{2}b^{2}\xi -4b\xi C_{L}+\beta^{2}C_{L}\right)}^{2}}) \end{array}$$

By calculating the first derivative function of ${\underset{\left(MS\right)}{\pi }}_{{}_{m}}^{TPT}$ with respect to $w_{MS}^{TPT}$, and letting $\frac{\partial {\underset{\left(MS\right)}{\pi }}_{{}_{m}}^{TPT}}{\partial w_{MS}^{TPT}}=0$, we can obtain the following expression:

(A25) $$\begin{array}{l} w_{{}^{MS}}^{TPT}=\frac{1}{4}\frac{\left(-8\xi^{2}b^{2}c_{m}+2\beta^{4}c_{m}-4\xi a\beta^{2}\right)C_{L}^{2}+4\Delta^{2}b\left(\xi \left(\frac{1}{2}\beta^{2}c_{m}+\xi a\right)b-\frac{1}{4}\beta^{4}c_{m}+\frac{1}{2}a\xi \beta^{2}\right)C_{L}-\Delta^{4}\xi ab^{2}\beta^{2}}{\left(\Delta^{2}b-2C_{L}\right)\left(\left(\xi^{2}b^{2}+\frac{1}{2}\xi b\beta^{2}-\frac{1}{4}\beta^{4}\right)C_{L}-\frac{1}{4}\Delta^{2}\xi b^{2}\beta^{2}\right)}\\ s.t.F_{MS}^{TPT}=\frac{4C_{L}{}^{2}{\left((a-bw_{MS}^{TPT})\xi +\frac{1}{2}(w_{MS}^{TPT}-c_{m})\beta^{2}\right)}^{2}}{b{\left(4\xi C_{L}-\Delta^{2}\beta^{2}\right)}^{2}}-\pi_{r}{}^{d*}(MS) \end{array}$$

In the same way, we can obtain the following expressions:

(A26) $$\tau_{MS}^{TPT}=\frac{\Delta (2bw_{MS}^{TPT}\xi -w_{MS}^{TPT}\beta^{2}+\beta^{2}c_{m}-2\xi a)}{\Delta^{2}\beta^{2}-4\xi C_{L}}$$

(A27) $$e_{m}{}_{MS}^{TPT}=\frac{\beta (\Delta^{2}w_{MS}^{TPT}b-\Delta^{2}a-2w_{MS}^{TPT}C_{L}+2c_{m}C_{L})}{\Delta^{2}\beta^{2}-4\xi C_{L}}$$

(A28) $$p_{MS}^{TPT}=\frac{[(w_{MS}^{TPT}-c_{m})\beta^{2}+2\xi (bw_{MS}^{TPT}+a)]C_{L}-\Delta^{2}b\beta^{2}w_{MS}^{TPT}}{\left(-\Delta^{2}b\beta^{2}+4\xi bC_{L}\right)}$$

□
